# Metabolic Control of Patients with Phenylketonuria in a Portuguese Metabolic Centre Comparing Three Different Recommendations

**DOI:** 10.3390/nu13093118

**Published:** 2021-09-06

**Authors:** Viviane Kanufre, Manuela Ferreira Almeida, Catarina Sousa Barbosa, Carla Carmona, Anabela Bandeira, Esmeralda Martins, Sara Rocha, Arlindo Guimas, Rosa Ribeiro, Anita MacDonald, Alex Pinto, Júlio César Rocha

**Affiliations:** 1 Centro de Genética Médica, Centro Hospitalar Universitário do Porto (CHUPorto), 4099-028 Porto, Portugal; vikanufre@gmail.com (V.K.); manuela.almeida@chporto.min-saude.pt (M.F.A.); catarina-s-@hotmail.com (C.S.B.); carla.carmona@chporto.min-saude.pt (C.C.); 2 Centro de Referência na Área de Doenças Hereditárias do Metabolismo, Centro Hospitalar Universitário do Porto-CHUPorto, 4099-001 Porto, Portugal; anabela.ol.bandeira@sapo.pt (A.B.); esmeralda.g.martins@gmail.com (E.M.); saraisabelrocha@gmail.com (S.R.); arlguimas@gmail.com (A.G.); rocrff@gmail.com (R.R.); 3 Núcleo de Ações e Pesquisa em Apoio Diagnóstico (NUPAD), School of Medicine, Federal University of Minas Gerais (UFMG), Avenida Professor Alfredo Balena, 190, Belo Horizonte 30130-100, Brazil; 4 Hospital das Clínicas, UFMG, Avenida Professor Alfredo Balena, 110, Santa Efigênia, Belo Horizonte 30130-100, Brazil; 5 Unit for Multidisciplinary Research in Biomedicine, Abel Salazar Institute of Biomedical Sciences, University of Porto-UMIB/ICBAS/UP, 4050-313 Porto, Portugal; 6 Birmingham Women’s and Children’s Hospital, Birmingham B4 6NH, UK; anita.macdonald@nhs.net (A.M.); alex.pinto@nhs.net (A.P.); 7 Nutrition & Metabolism, NOVA Medical School, Faculdade de Ciências Médicas, Universidade Nova de Lisboa, Campo Mártires da Pátria, 130, 1169-056 Lisbon, Portugal; 8 Centre for Health Technology and Services Research (CINTESIS), 4200-450 Porto, Portugal

**Keywords:** phenylketonuria, phenylalanine, metabolic control, guidelines

## Abstract

Blood phenylalanine (Phe) is used as the primary marker to evaluate metabolic control. Our study aimed to describe the metabolic control of patients with phenylketonuria (PKU) comparing three different treatment recommendations (European guidelines/US guidelines/Portuguese consensus). This was a retrospective, observational, single centre study in patients with PKU collecting data on blood Phe levels from 2017. Nutritional intake data and sapropterin (BH4) prescription were collected at the last appointment of 2017. The final sample studied included 87 patients (48% females) [13 hyperphenylalaninemia; 47 mild PKU; 27 classical PKU] with a median age of 18 y (range: 1–36 y). The median number of blood Phe measurements for patients was 21 (range: 6–89). In patients aged < 12 y, the median blood Phe level was 300 μmol/L (range 168–480) and 474 μmol/L (range 156–1194) for patients ≥ 12 y. Overall, a median of 83% of blood Phe levels were within the European PKU guidelines target range. In patients aged ≥ 12 years, there was a higher median % of blood Phe levels within the European PKU guidelines target range (≥12 y: 84% vs. <12 y: 56%). In children < 12 y with classical PKU (*n =* 2), only 34% of blood Phe levels were within target range for all 3 guidelines and 49% with mild PKU (*n =* 11). Girls had better control than boys (89% vs. 66% median Phe levels within European Guidelines). Although it is clear that 50% or more patients were unable to achieve acceptable metabolic control on current treatment options, a globally agreed upper Phe target associated with optimal outcomes for age groups is necessary. More studies need to examine how clinics with dissimilar resources, different therapeutic Phe targets and frequency of monitoring relate to metabolic control.

## 1. Introduction

Phenylketonuria (PKU) is an inborn error of amino acid metabolism characterized by persistent hyperphenylalaninemia due to a deficiency of the hepatic phenylalanine hydroxylase enzyme. This prevents the hydroxylation of the essential amino acid phenylalanine (Phe) into tyrosine (Tyr) leading to increased blood and brain Phe concentrations. Immediate and sustained treatment following newborn screening is crucial to enable normal development, health and well-being throughout life [1]. While high blood Phe concentrations during childhood are known to primarily affect intellectual functioning, increased levels during adulthood are associated with neurological, mental health, executive functioning and behavioural problems, as well as deficits in social skills [2].

The main treatment objective is to maintain blood Phe levels within a safe target therapeutic range, while providing necessary macro and micronutrients to enable proper growth and development [3]. The core treatment is a Phe restricted diet (by restriction of natural protein), supplemented with a low/free Phe protein substitute (PS) and special low protein foods (SLPFs) [3,4,5,6]. The availability of special medical foods for PKU varies widely across countries [5,6], potentially influencing blood Phe control. The dose (g/kg) and protein source of protein substitutes also impacts metabolic control [3,7,8,9]. It is well recognized that adhering to a Phe restricted diet is particularly challenging [10,11]. 

A subgroup of patients with PKU, usually with a milder PKU phenotype may benefit from a drug therapy, tetrahydrobiopterin (BH4) (sapropterin dihydrochloride). BH4 acts as a pharmaceutical chaperone increasing the residual activity of the phenylalanine hydroxylase enzyme; BH4 responsive patients are generally able to increase their natural protein intake by 2 to 4-fold and/or reduce blood Phe levels [12]. Pegvaliase, the newest treatment option, is an enzyme substitution therapy that converts Phe to trans-cinnamic acid and ammonia, and was approved in the United States (US) in 2018 and Europe in 2019 [13]. It is administered by subcutaneous injection, and it is effective in lowering blood Phe levels and increasing Phe tolerance. However, pegvaliase may be associated with immunologic reactions, requiring careful management [13]. This treatment is directed at adults with blood Phe levels ≥ 600 µmol/L and is not recommended in pregnancy. Further studies are required looking at its long-term efficacy and safety. 

Blood Phe is used as the primary marker to evaluate metabolic control. Routine blood Phe is the only practical marker even though the aim of treatment is to prevent high Phe levels in the brain due to Phe transport across the blood brain barrier [1]. This is particularly important in early life when high Phe levels have a severe impact in brain and neurological development [14,15,16]. However, chronic long term high blood Phe levels in adulthood may also impact cognition and there are an increasing case studies of people with PKU developing neurological issues with increasing age [17,18,19]. International PKU guidelines give recommendations for target blood Phe levels which are essential to guide and monitor treatment, assess patient’s outcomes, and compare effectiveness of treatments [1,20]. 

International guidelines representing different nations have recommended different target therapeutic ranges for blood Phe levels and there is conflict amongst professionals regarding the optimal target range, particularly in adult patients with PKU [21]. In 2017, the European PKU Guidelines gave scientific evidence to support an upper blood Phe target level of 360 μmol/L for children aged < 12 y (years) and for patients aged ≥ 12 y, 600 μmol/L [1]. The United States (US) 2014 guidelines recommended a target blood Phe level of 360 μmol/L for all age groups [20]. In 2007, in Portugal, a PKU working group from the Portuguese Society for Metabolic Disorders (SPDM) suggested an upper Phe target in the Portuguese Consensus of 360 μmol/L up to 12 y and 480 μmol/L onwards [22]. A recent publication from Portugal showed that professionals did not agree with all the key statements from the European PKU Guidelines [23]. Although 100% of professionals agreed with target blood Phe levels for patients < 12 y, this decreased to only 32% for the recommendations ≥ 12 y [23]. In practice, Portuguese centres already aimed for a lower ‘upper’ blood Phe target level (480 μmol/L) and were uncomfortable relaxing this to a maximum upper limit of 600 μmol/L without conclusive evidence. 

This study aims to describe the metabolic control of patients with PKU in a single Portuguese centre comparing three different recommendations (European guidelines, US guidelines and Portuguese consensus) [1,20,22].

## 2. Materials and Methods 

### 2.1. Participants

All patients with PKU being treated at Centro Hospitalar Universitário do Porto were considered for this study (*n =* 136). 

The severity of the disorder was classified according to neonatal blood Phe levels at newborn screening, as defined by the Portuguese Consensus: hyperphenylalaninemia (blood Phe < 360 μmol/L), mild PKU (blood Phe ≥ 360 and ≤1200 μmol/L), and classical PKU (blood Phe > 1200 μmol/L) [22]. 

Exclusion criteria included: failure to attend clinic appointments, <6 blood Phe measurements in the year of study, late diagnosed with PKU and pre-conception diet/pregnancy during the 12-month study period.

### 2.2. Study Design

This was a retrospective, observational, single centre study about blood Phe control of patients with PKU. All blood Phe and Tyr levels were taken during 2017. Nutritional intake data and BH4 prescription were also collected at the last appointment of 2017. All data were collected from electronic patient clinical records. 

### 2.3. Data Collection

#### 2.3.1. Nutritional Intake

Dietary intake data were collected for natural protein (NP; g/kg/day), protein equivalent from protein substitute (PS; g/kg/day) and total protein (TP; g/kg/day). Twenty-four hour dietary recalls were performed by experienced nutritionists (M.F.A. and J.C.R.) at each clinic to assess dietary intake. These data were transferred to an Excel sheet (Microsoft, Washington, DC, USA) which calculated nutritional intake. This excel is formatted with the nutritional composition from the Portuguese Food Composition Tables for normal foods and composition of the SLPFs and PS available in Portugal. 

The three main treatment types used were defined in our analysis:

- **PKU diet only**: Phe restricted diet supplemented with PS and SLPFs
- **BH4 + diet:** BH4 treated patients with Phe restriction and ±PS
- **Non-restricted diet:** without PS or BH4 prescription

#### 2.3.2. BH4 

In patients taking BH4, data were collected on dose prescribed in mg/kg. Kuvan^®^ from Biomarin was the BH4 molecule prescribed.

#### 2.3.3. Metabolic Control

Blood Phe levels were measured from fasting dried blood spots taken by patients/caregivers and analyzed using a tandem mass spectrometry. Patients/caregivers were instructed by a nurse about the dried blood spot taking technique.

Data, stored on the patient database, were collected by a dietetic researcher (V.K.). Median blood Phe and Tyr levels were calculated and % of annual blood Phe measurements within target range from the year of data collection. Frequency of recommended monitoring was once weekly until 1 y, once every 2 weeks until 12 y and once monthly ≥ 12 y [20]. 

Blood Phe levels were compared with the European PKU Guidelines (recommended blood Phe levels 120–360 μmol/L up to 12 y and 120–600 μmol/L onwards) [1], US PKU guidelines (120–360 μmol/L throughout life) [20] and Portuguese Consensus (blood Phe levels 120–360 μmol/L up to 12 y and 120–480 μmol/L onwards) [22].

### 2.4. Ethical Statement

The study protocol was approved by the ethical committee of Centro Hospitalar Universitário do Porto on the 19 December 2018 (Reference 2018.199). Written informed consent was obtained from either each patient or caregiver (according to age). Participants were identified by a code to maintain patient anonymity.

### 2.5. Statistical Analysis

Descriptive statistics were used to present the results. Categorical variables were presented as absolute values or percentages, while continuous variables were presented as medians. 

## 3. Results

### 3.1. Study Cohort

From 136 patients followed up in clinic, 49 patients were excluded due to: no attendance to scheduled appointments, either no blood Phe measurements during 2017 (*n =* 18) or <6 blood Phe measurements during the study period (*n* = 16); late diagnosed patients (*n =* 12); and pre-conception diet/pregnancy (*n =* 3). 

The final sample studied included 87 patients (48% females) with a median age of 18 y (range from age 1 to 36 y). Nineteen patients were <12 y (median age of 8 y; range 1–11 y) and 68 patients ≥ 12 y (median age 22 y; range 12–36 y). Of the 68 patients ≥ 12 y, 36 patients (53%) were >20 y. 

There were 13 patients with hyperphenylalaninemia (15%), 47 with mild PKU (54%), and 27 patients with classical PKU (31%).

Table 1 presents patients characteristics by age, gender, disorder severity and type of treatment prescribed.

### 3.2. Nutritional Intake

Patients were prescribed three main types of treatment; (1) PKU diet only, which is a Phe restricted diet supplemented with PS and SLPFs, *n* = 50; (2) BH4 (BH4 treated patients with Phe restriction and ±PS), *n =* 22; and (3) a non-restricted diet (without PS or BH4 prescription), *n =* 15. Table 2 presents nutritional protein intake regarding age, disorder severity and type of treatment prescribed.

Of 50 patients prescribed a Phe restricted diet, *n* = 41 were given Phe-free amino acids, *n =* 4 Phe-free amino acid together with glycomacropeptide (CGMP-AA); and 5 patients CGMP-AA supplement only. For the patients on long term BH4 treatment (*n =* 22), (duration of BH4 treatment = median 2 years), 18 required supplementation with a PS. The median daily dose of BH4 was 15.5 mg/kg/day (range 11.6–20.6 mg/kg). 

Patients in the non-restricted diet group (*n* = 15) all met safe levels of protein intake [21] without the use of PS.

### 3.3. Metabolic Control—Portuguese Consensus 

The median number of blood Phe measurements for each patient recorded in 2017 was 21 (range 6–89). In patients aged < 12 y, the median blood Phe level was 300 μmol/L (range 168–480); blood Tyr was 71 μmol/L (range 43–96). In patients aged ≥ 12 years, the median blood Phe level was 474 μmol/L (range 156–1194) and Tyr was 67 μmol/L (range 40–94). Median results were within the Portuguese targets for both age groups [22]. However, in children < 12 years with mild PKU, only 49% of levels were within target range. Girls had overall better control than boys (median % of blood Phe levels within target range was females: 66% (aged ≥ 12 y) and 74% (aged < 12 y); males: 41% (aged ≥ 12 y) and 45% (aged < 12 y)). When assessing the younger and older age groups, the percentage of blood Phe within target range improved with age in the mild PKU group and remained unchanged in the classical and HPA group.

Table 3 presents the median % of blood Phe levels within target range, recommended by the Portuguese consensus [22] stratified by age, sex, disease severity and type of treatment.

**Figure 1:**
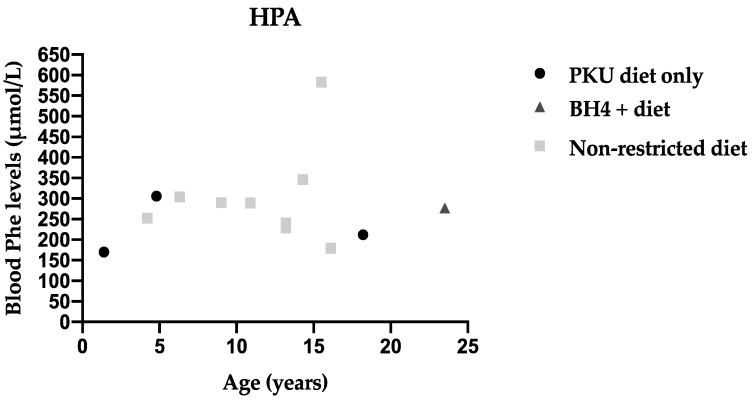
Median annual blood Phe levels for each individual patient with HPA by age and type of treatment.

**Figure 2:**
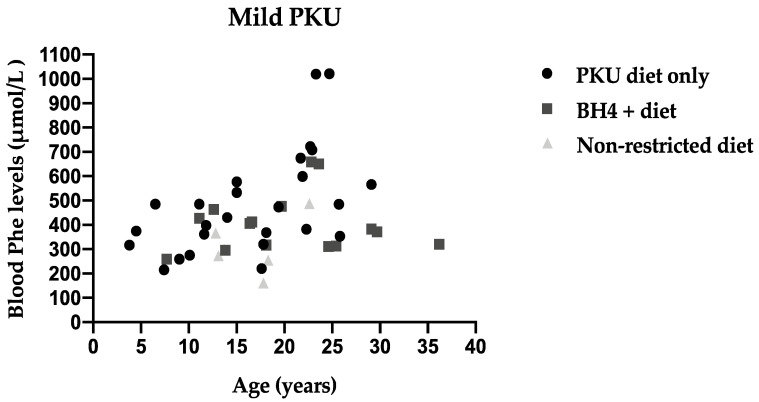
Median annual blood Phe levels for each individual patient with mild PKU by age and type of treatment.

**Figure 3:**
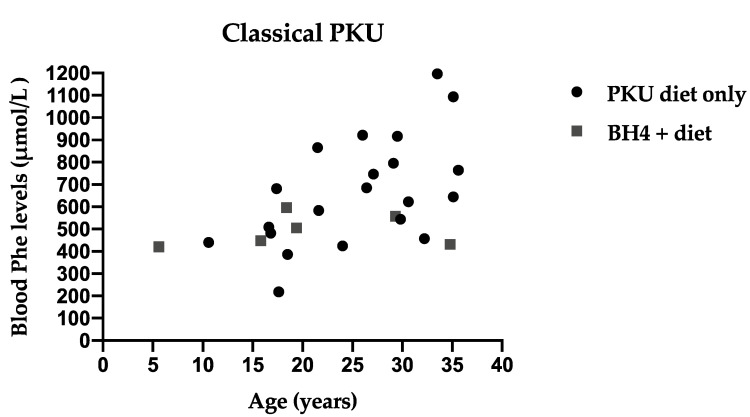
Median annual blood Phe levels for each individual patient with Classical PKU by age and type of treatment.

Annual median blood Phe levels are presented for each patient by age and type of treatment in HPA patients (Figure 1), Mild PKU (Figure 2) and Classical PKU (Figure 3).

### 3.4. Metabolic Control Comparing Three Different Recommendations

**Figure 4:**
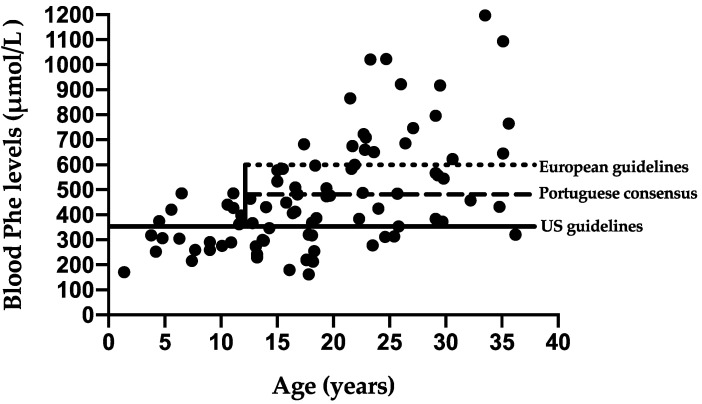
Median annual blood Phe levels presented for each patient compared to three different recommendations.

Annual median blood Phe levels increased with age. Figure 4 shows the annual median of blood Phe levels for each patient studied, comparing to the upper target levels of the Portuguese consensus, European and US guidelines. 

The median percentage of blood Phe levels within target range according to the two International guidelines and the Portuguese consensus are given in Table 4. 

## 4. Discussion

This study demonstrated that patients with classical PKU struggled to achieve an acceptable level of blood Phe control on dietary treatment only, irrespective of age or the upper target blood Phe level guideline. Their blood Phe control was suboptimal compared with mild PKU and HPA. These figures most likely underestimate poor control, as they do not consider the excluded patients from this study who either did not attend clinic appointments or failed to return blood Phe spots. Overall, this was a group of patients who were well supported by their clinical multidisciplinary team (nutritionist, psychologist, clinician). Patients with classical PKU only tolerated 0.4 g/kg (range: 0.17–1.80) of natural protein and a total protein intake of 1.37 g/kg (range: 0.95–1.80). It is possible that a higher dose of PS may have improved blood Phe control as their total protein intake was lower than the other 2 groups, although safe levels of protein intake were met [24]. 

Patients with classical PKU may have been unable to maintain their severe and onerous dietary restriction. Not only is the Phe restricted diet very limited, but it also involves maintaining strict dietary routines, preparation of SLPFs, planning daily Phe consumption, preparing low-Phe meals and meticulously planning every activity that involves food. It incurs a time management burden of 19 h per week [22]. Moreover, maintaining a Phe restrictive diet seems even more challenging than in the past. Some protein substitutes such as CGMP-AA contain Phe which may complicate gaining acceptable blood Phe control in children [7]. Persistent consumer pressure from the food industry and busy working adult lives has led to increased dependence on processed foods which are commonly not low in Phe or may have unreliable protein labelling information [25]. In addition, societal efforts to reduce the sugar content of foods has also led to sugar replacement by artificial sweeteners such as aspartame, another unquantified source of Phe [26]. Pre-existing social disadvantages such as parental poor literacy, health literacy and poverty may render some children particularly vulnerable. 

The evidence to support an upper blood Phe level of 360 µmol/L in children aged under <12 y is convincing and is supported by all three PKU Guidelines/Consensus [1,20,22]. Therefore, the low percentage of blood Phe levels within target range for children aged < 12 y was a concern. Even children with mild PKU achieved <50% of blood Phe levels < 360 µmol/L. There is much evidence to suggest that the inability to sustain good metabolic control in childhood is associated with a decline in IQ (intelligence quotient) score and executive function and will have a negative influence in adulthood [14,17,18]. A meta-analysis estimated that an increase of 100 μmol/L in lifetime Phe levels predicts an average 1.9 to 4.1 point reduction in IQ over a range of Phe from 394 to 666 μmol/L [15]. Jaha et al. in 2017 showed that high blood Phe levels in childhood, affect adult cognitive flexibility, executive motor control, executive function in daily life and adult mental health [17]. Weglage et al. (2013) also showed that high blood Phe levels in childhood and adolescence were related to poorer IQ, information processing and attention in adulthood [27]. It is evident that alternative treatment choices are necessary to help improve the control of this group of patients with PKU. Much attention is directed at identifying effective non dietary treatments for adults but is essential that the paediatric population is not neglected. However, even when children were on BH4, only 49% had blood Phe levels within the target therapeutic range. 

Deterioration of blood Phe control is well described with age. However, for our patient cohort, the overall % of blood Phe levels within the target therapeutic range (Portuguese consensus) did not deteriorate in patients aged ≥ 12 y. In fact, over 80% of Phe levels were within the European PKU target range but only 17% below the US guideline upper target range. Older patients with BH4 treatment for 2 y duration benefited from a relaxed dietary treatment without loss of metabolic control. Overall, the differences in upper blood Phe target ranges between local recommendations, Europe and USA are confusing and unsatisfactory for both patients and health professionals, particularly when the same evidence-based approach has been used to develop the two different guidelines. Both upper target levels aim to maintain safety of adult patients and prevent neurological and mental health complications, but neither recommendation is supported by robust clinical studies. 

Even so, evidence is accumulating that significant sub-optimal outcomes exist in early treated adult patients. Pilotto et al. (2020) [28] provided evidence from 19 adult patients (median blood Phe level 873 μmol/L) showing that blood Phe levels were highly correlated with the number of failed neuropsychological tests, neuropsychiatric symptoms, motor evoked potential latency and parietal lobe atrophy high and there was direct association between brain function and metabolic control in adulthood. Historically diet was discontinued in many children and teenagers, and there is evidence of neurological symptoms in some patients [29]. Early treated patients with PKU have only reached 50 y, and little is known about their aging process in later adulthood. Due to this uncertainty of outcome and historical errors and missteps that have been made over treatment duration and degree of metabolic control, it is unsurprising that some international guidelines suggest stricter metabolic control for their adult populations. 

We consider that the focus should be on seeking alternative treatments and home monitoring tools to help self-care and alleviation of strict dietary treatment. Treatments associated with minimal side effects, that are easy to administer and associated with optimal neurocognitive and mental health outcomes are essential. More resources/tools are needed to allow patients to achieve the lowest blood Phe level within target therapeutic range with no negative impact on quality of life. This is especially needed for classical patients with PKU who particularly struggle to meet the defined targets. These patients face bigger challenges with much lower Phe tolerances compared to other patient subgroups. 

There are several limitations in this project. This is a retrospective uncontrolled study, only reflecting results of routine clinical practice for 1 year only in a single clinic. Protein intake in patients on a non-restrictive diet was not controlled as in other patients. Blood Phe levels are associated with error, reflective of blood specimen quality and concentration [30]. Blood Phe levels may also not directly reflect neurotransmitter metabolism. Exclusion of patients who did not attend clinic or perform blood samples may have altered the median percentage of blood Phe results observed. Also, the number of blood spots returned varied between patients. Twenty-four hours dietary recalls used for assessing dietary intake are associated with error and inaccuracy. 

## 5. Conclusions

In general, blood Phe levels were around 56% within therapeutic target according to the Portuguese consensus although there is a tendency for increasing median blood Phe levels with age. The number of blood Phe levels within target range according to the European guidelines and US guidelines blood Phe levels were around 83% and 26%, respectively. In consideration of the different Phe upper limits recommended, we must strive for safe levels that are associated with the best patient outcomes. There should be focus on improving alternative treatment options and clinical resources to enable patients to achieve lower blood Phe levels. More studies are needed comparing outcome of centres using different blood Phe targets, their frequency of monitoring and the resources that they have available to them to determine optimal blood Phe control. 

## Figures and Tables

**Table 1:** Patient’s characteristics by type of treatment, gender, age and severity of PKU.

Variable		<12 Years *n* (%)	≥12 Years *n* (%)	Total *n* (%)
**N**		19 (22)	68 (78)	87 (100)
**Gender**	**Female**	8 (42)	34 (50)	42 (48)
	**Male**	11 (58)	34 (50)	45 (52)
**PKU severity**	**Classical PKU**	2 (11)	25 (37)	27 (31)
	**Mild PKU**	11 (58)	36 (53)	47 (54)
	**HPA**	6 (32)	7 (10)	13 (15)
**Type of treatment**	**PKU diet only**	12 (63)	38 (56)	50 (57)
	**BH4 + diet**	3 (16)	19 (28)	22 (26)
	**Non-restricted diet**	4 (21)	11 (16)	15 (17)

**Abbreviations: HPA**: hyperphenylalaninemia; **PKU**: Phenylketonuria; **BH4**: sapropterin; **PKU diet only**: Phenylalanine restricted diet supplemented with protein substitute and special low protein foods; **BH4 + diet**: BH4 treatment with Phe restriction and ±protein substitute; **Non-restricted diet:** without protein substitute or BH4.

**Table 2:** Median intake of natural protein, protein equivalent and total protein.

Variable		Median Natural Protein (P25–P75) g/kg/Day	Median Protein Equivalent (P25–P75) g/kg/Day	Median Total Protein (P25–P75) g/kg/Day
**Total**		0.69 (0.12–4.09)	0.74 (0.00–1.55)	1.54 (0.68–4.09)
**Age**	**<12 y (*n =* 19)**	0.69 (0.28–4.09)	0.89 (0.00–1.55)	1.84 (1.23–4.09)
	**≥12 y (*n =* 68)**	0.69 (0.12–2.55)	0.72 (0.00–1.32)	1.46 (0.68–2.55)
**PKU** **Severity**	**Classical PKU (*n =* 27)**	0.40 (0.17–1.80)	0.85 (0.00–1.32)	1.37 (0.95–1.80)
	**Mild PKU (*n =* 47)**	0.69 (0.12–2.4)	0.74 (0.00–1.55)	1.54 (0.68–2.40)
	**HPA (*n =* 13)**	1.97 (1.39–4.09)	0.00 (0.00–0.51)	2.10 (1.60–4.09)
**Type of treatment**	**PKU diet only (*n =* 50)**	0.48 (0.17–1.80)	0.87 (0.08–1.55)	1.47 (0.95–3.60)
	**BH4 + diet (*n =* 22)**	0.99 (0.24–1.84)	0.63 (0.00–1.07)	1.53 (0.68–2.19)
	**Non-restricted diet (*n =* 15)**	1.97 (1.26–4.09)	0.00 (0.00–0.00)	1.97 (1.26–4.09)

**Abbreviations: HPA**: hyperphenylalaninemia; **PKU**: Phenylketonuria; **BH4**: sapropterin; **PKU diet only**: Phenylalanine restricted diet supplemented with protein substitute and special low protein foods; **BH4 + diet**: BH4 treatment with Phe restriction and ±protein substitute; **Non-restricted diet:** without protein substitute or BH4.

**Table 3:** Median percentage of blood Phe measurements within target range recommended by the Portuguese consensus.

		<12 Years	≥12 Years
		Median % of Blood Phe Levels within Target Range *	Median % of Blood Phe Levels within Target Range *
**Sex**	**Female (*n =* 42)**	74	66
	**Male (*n =* 45)**	45	41
**PKU severity**	**Classical PKU (*n =* 27)**	34	28
	**Mild PKU (*n =* 47)**	49	77
	**HPA (*n =* 13)**	91	100
**Type of treatment**	**PKU diet only (*n =* 50)**	54	27
	**BH4 + diet (*n =* 22)**	49	84
	**Non-restricted diet (*n =* 15)**	73	100

**Abbreviations: HPA**: hyperphenylalaninemia; **PKU**: Phenylketonuria; **BH4**: sapropterin; **PKU diet only**: Phenylalanine restricted diet supplemented with protein substitute and special low protein foods; **BH4 + diet**: BH4 treatment with Phe restriction and ±protein substitute; **Non-restricted diet**: without protein substitute or BH4. * Portuguese consensus.

**Table 4:** Median percentage of blood Phe measurements within target range according to the Portuguese consensus, European and US guidelines.

		Median % of Blood Phe Levels within Target Range		
	Variable	Portuguese Consensus % (p25–p75)	European Guidelines % (p25–p75)	US Guidelines % (p25–p75)
	**Total**	56 (19–94)	83 (36–100)	26 (0–78)
**Age**	**<12 years (*n =* 19)**	56 (23–87)	56 (23–87)	56 (23–87)
	**≥12 years (*n =* 68)**	54 (13–96)	84 (38–100)	17 (0–60)
**Gender**	**Female (*n =* 42)**	70 (23–98)	89 (42–100)	29 (0–81)
	**Male (*n =* 45)**	41 (13–89)	66 (22–99)	22 (0–59)
**PKU severity**	**HPA (*n =* 13)**	100 (84–100)	100 (85–100)	96 (7–100)
	**Mild PKU (*n =* 47)**	63 (19–94)	84 (39–84)	41 (0–76)
	**Classical PKU (*n =* 27)**	27 (0–44)	47 (4–83)	6 (0–26)
**Type of treatment**	**PKU diet only (*n =* 50)**	32 (8–74)	59 (14–91)	15 (0–51)
	**BH4 diet (*n =* 22)**	76 (44–92)	91 (66–100)	32 (17–76)
	**Non-restricted diet (*n =* 15)**	97 (33–100)	100 (83–100)	83 (13–100)

**Abbreviations: US:** United States; **HPA**: hyperphenylalaninemia; **PKU**: Phenylketonuria; **BH4**: sapropterin; **PKU diet only**: Phenylalanine restricted diet supplemented with protein substitute and special low protein foods; **BH4 + diet**: BH4 treatment with Phe restriction and ±protein substitute; **Non-restricted diet:** without protein substitute or BH4.

## Data Availability

The data will be made available from the authors upon reasonable request.
